# Research progress on the synergistic regulation of MYB transcription factor-mediated developmental plasticity and stress responses in rice

**DOI:** 10.3389/fpls.2025.1668800

**Published:** 2025-10-08

**Authors:** Wenyan Peng, Yi Zhang, Hongjun Xie, Yinghong Yu, Mingdong Zhu

**Affiliations:** ^1^ College of Biology, Hunan University, Changsha, China; ^2^ State Key Laboratory of Hybrid Rice, Hunan Academy of Agricultural Sciences, Changsha, China; ^3^ Yuelushan Laboratory, Changsha, China

**Keywords:** MYB transcription factor, rice, synergistic regulation, developmental plasticity, stress response

## Abstract

As a global staple crop, rice production is seriously threatened by biotic and abiotic stresses. OsMYB, which is a core transcriptional regulator in plants, mediates key processes, including secondary metabolism, organ development, and stress responses. In this paper, we systematically describe the mechanisms underlying the synergistic effects of OsMYB family members on developmental plasticity and stress adaptations. Specifically, 1R-MYB transcription factors mainly regulate metabolism and yield formation, R2R3-MYB transcription factors mainly regulate organ development and stress resistance through the bidirectional modulation of the antioxidant system and lignin synthesis, 3R-MYB transcription factors primarily affect cell cycle regulation, and 4R-MYB transcription factors have functions that remain to be characterized. Further elucidating OsMYB-mediated molecular networks may lead to new strategies for developing stress-resistant and high-yielding rice varieties, with positive implications for sustainable agricultural development.

## 1 Introduction

Rice (*Oryza sativa L.*), which is one of the most important food crops in China, is also the main source of food for nearly half of the global population (Zhang et al., 2014). Thus, a high and stable rice yield is crucial for ensuring food security worldwide. Recent global climate change-induced warming (Feng et al., 2022; Su et al., 2025), extreme precipitation (Wei et al., 2024), changes in the western flank of subtropical high pressure (Bregy et al., 2025), and increasing atmospheric CO_2_ concentrations with increasing carbon emissions (Liu et al., 2018b; Tu et al., 2024) represent unprecedented and complex challenges to rice breeding and production.

Multiple climate change-related abiotic stresses have synergistic effects on crop production, with rice often simultaneously exposed to several stressors, including high temperatures combined with drought conditions (Zhan et al., 2023) or flooding accompanied by pest infestations and pathogen infections (He et al., 2023), during its growth cycle. For example, temperatures exceeding 35°C for 5 days during flowering lead to sterility and a lack of seed setting in spikelets (Jagadish et al., 2007), while intermittent drought conditions further exacerbate the harmful effects of water stress. Hence, new varieties must contain heat tolerance-related genes and exhibit drought resistance characteristics. Additionally, central, eastern, and northwestern regions in China may be severely affected by climate change, with rice yields decreasing by 41.5% (Zhan et al., 2023).

In terms of biotic stresses, climate change has resulted in favorable environmental conditions for pests and pathogens, leading to enhanced reproduction and increases in the number of pests and pathogens (Fu et al., 2010). Moreover, pests (e.g., *Plutella xylostella*) are increasingly becoming resistant to chemical pesticides (Ma et al., 2021), thereby necessitating the breeding of varieties with broad-spectrum durable resistance and decreased dependence on pesticides. This may be accomplished by aggregating multiple pest and disease resistance genes and applying ecological control measures. Accordingly, modern rice breeding programs must focus on varietal adaptability to the synergistic effects of multiple environmental stresses. They must also modulate various agronomic traits influencing crop quality and stress resistance and develop new varieties that can adapt to environmental conditions and produce high and stable yields.

Transcription factors are critical regulators of plant growth, development, and stress responses. More specifically, they are major components of molecular mechanisms underlying stress signal transduction in rice (Baldoni et al., 2015). As trans-acting proteins, transcription factors bind specifically to cis-acting elements in the promoter regions of eukaryotic genes. These proteins can bind to DNA and precisely regulate target gene transcription by interacting with cis-elements and other proteins.

On the basis of sequence features and similarities in DNA-binding domain structures, plant transcription factors have been systematically classified into various families (Pabo and Sauer, 1992). These include WRKY (transcription factors, TFs), MYB (v-myb avian myeloblastosis viral oncogene homolog), DREB (drought response element binding), NAC [NAM (no apical meristem), ATAF1/2 (Arabidopsis transcription activation factors), CUC2 (cup-shaped cotyledon 2)], zinc-finger proteins, bZIP (basic leucine zipper), AP2/ERF (APETALA2/ethylene response factor), CBF (C-repeat binding factor), and bHLH (basic helix-loop-helix). Notably, MYB proteins belong to one of the largest transcription factor families in plants (Wu et al., 2024b). They play important roles in key developmental processes, such as cell differentiation (Lipsick, 1996) and morphogenesis (Yang et al., 2014a; Piao et al., 2019; Wang et al., 2022), while also affecting multiple physiological processes, including responses to abiotic stresses (e.g., high temperature, drought, and high salinity) (El-Kereamy et al., 2012; Yang et al., 2012; Tang et al., 2019) and adaptations to biotic stresses (e.g., pathogen defense) (Kishi-Kaboshi et al., 2018).

Moreover, MYB transcription factors modulate plant growth, development, and stress responses by forming complex synergistic regulatory networks with other transcription factors, including WRKY, bZIP, and NAC family members. These interactions involve the activation, inhibition, and fine-tuning of target gene expression, enabling plants to respond efficiently and precisely to internal and external signals.

MYB transcription factors collaborate with other regulators in various ways. For example, MYB activators and inhibitors competitively bind to the same promoter elements and interact with third-party factors (e.g., WRKY) to precisely control the timing of downstream gene expression (Mu et al., 2023). Many synergistic effects are mediated through direct protein interactions, including those between bZIP44 and MYB10/MYB72 (Wu et al., 2024a). Additionally, MYB22 recruits TOPLESS and HDAC1 via its EAR motif to form a repressive complex (Sun et al., 2023).

This review comprehensively summarizes the molecular regulatory networks and mechanisms underlying the effects of MYB transcription factors on rice growth, development, and stress responses. The following sections focus on the core regulatory functions of MYB family members influencing morphogenesis and reproductive development as well as their molecular interactions within defense systems during responses to biotic and abiotic stresses. This review provides information regarding key target genes and molecular design strategies for enhancing the molecular regulatory framework of rice, with possible implications for breeding new stress-resistant and high-yielding varieties.

## 2 MYB transcription factor types and modes of action

As one of the most important regulatory proteins in plants, MYB transcription factors are molecularly characterized by a conserved N-terminal MYB DNA-binding domain (MYB domain) comprising 50–52 amino acids (Rosinski and Atchley, 1998). This domain binds to regulatory elements of target genes through a sequence-specific recognition mechanism (Jin and Martin, 1999). Typical MYB transcription factors have specific tertiary structural characteristics: DNA-binding domain (DBD), transactivation domain, and negative regulatory domain. More specifically, DBD is composed of 1–4 incomplete MYB repeat units, each containing 50–52 amino acids, with three α-helix structures forming a helix-turn-helix topology in which the second and third helices interact specifically with the major groove of the DNA double helix via spatial conformational adaptation (Thompson and Ramsay, 1995; Ogata et al., 1996). This evolutionarily conserved DBD module determines the ability of MYB proteins to recognize target sequences, ultimately leading to the regulated expression of downstream genes that mediate plant developmental processes and stress response mechanisms.

**Figure 1:**
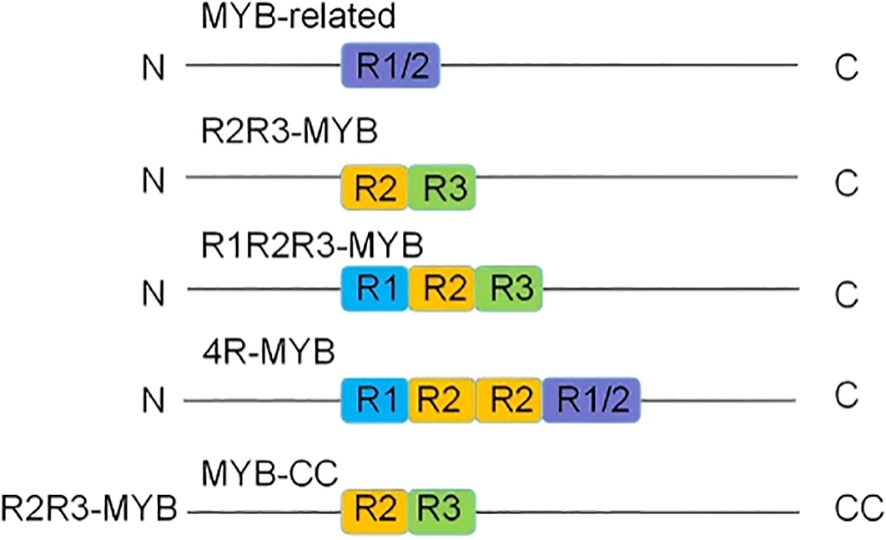
Classification of MYB transcription factors. On the basis of the number of MYB repeat sequences and their similarity to the prototype repeat sequence, MYB transcription factors have been divided into four subclasses: 1R-MYB/MYB-related (R1-MYB), 2R-MYB (R2R3-MYB), 3R-MYB (R1R2R3-MYB), and 4R-MYB (R1R2R3-MYB). MYB-CC transcription factors belong to a separate category.

According to MYB domain characteristics, plant MYB transcription factors can be divided into four evolutionary clades (**Figure 1**): 1R-MYB/MYB-related (R1-MYB) with a single MYB domain, 2R-MYB with two repeat units (R2R3-MYB), 3R-MYB with three repeat units (R1R2R3-MYB), and 4R-MYB (R1R2R2R1/R2-MYB) with four repeat units. Among these clades, 4R-MYB is the rarest (Dubos et al., 2010; Kang et al., 2022). To date, 197 MYB transcription factors have been identified in rice (Katiyar et al., 2012). Dicotyledon and monocotyledon genomes contain more than 100 R2R3-MYB gene family members, whereas the 1R-MYB and 3R-MYB gene families are smaller, each with fewer than five members (Dubos et al., 2010; Sun et al., 2019). Research on 4R-MYBs remains limited, and their functions have not been linked to plant stress resistance. This review summarizes the regulatory effects of four 1R-MYBs, 19 R2R3-MYBs, and three 3R-MYBs on rice growth and development. Additionally, five 1R-MYBs, 24 R2R3-MYBs, one 3R-MYB, and one MYB-CC that regulate abiotic stress responses as well as eight R2R3-MYBs that regulate biotic stress responses are described.

Notably, the R2R3-MYB subfamily has expanded significantly in terrestrial plants (Jiang et al., 2004). The molecular mechanism underlying its evolution has become a hot topic among researchers investigating plant transcription factors because it is closely related to its key regulatory roles in the plant kingdom (Jia et al., 2004). Although the functions of homologous R2R3-MYB proteins may differ among species, phylogenetic analyses have shown that they are more inclined to achieve sophisticated regulation of regulatory networks through functional synergy rather than simple gene redundancy (Jiang et al., 2004).

The phosphorus starvation response (PHR) 1 protein belongs to the MYB-CC family because of its unique structural domain. Members of this family have a MYB DNA-binding domain and a coiled-coil (CC) structural domain, which self-assembles via an α-helix to form a homo/heterodimeric complex; this type of protein interaction represents the structural basis for the regulatory functions of MYB-CC family members (Yang et al., 2024).

MYB proteins recognize specific DNA sequences through characteristic structural domains, including the R2R3-MYB repeat (Dubos et al., 2010), thereby enabling the precise regulation of multiple biological processes. The mechanism involves MYB domain-mediated binding to cis-acting elements in promoter regions of downstream target genes. Transcriptional complexes dynamically regulate target gene transcription, leading to the regulation of development and the integration of environmental signals in plants. This binding activity may be modulated through molecular interactions with upstream regulators, including co-activator or repressor proteins (Kobayashi et al., 2015), resulting in phenotypic plasticity. In addition, MYB family members can form functional complexes via homo/heteropolymerization, which can significantly increase their binding affinity and regulatory specificity toward target genes.

MYB transcription factors play a pivotal role in plant development and response to stress by constructing multidimensional molecular interaction networks. Their regulatory effects include, but are not limited to: (1) synergistic or independent regulation of physiological processes through the regulation of signaling pathways of phytohormones, including abscisic acid (ABA), auxin (IAA), and gibberellin (GA) (Yi et al., 2023; Kim et al., 2024); (2) participation in the mitogen-activated protein kinase (MAPK) cascade pathway, the reactive oxygen species signaling network, and the phospholipase D/phosphatidic acid (PLD/PA) signaling pathway (Zhu et al., 2015; Lin et al., 2022), thereby systematically enhancing plant adaptations to abiotic stresses.

In rice, MYB transcription factors represent important molecular regulatory hubs, with functions affecting the following: (1) growth and development: fine-tuning of the seed germination regulatory network (Wang et al., 2022), root conformation establishment (Piao et al., 2019), and leaf senescence and photoperiod-dependent flowering pathways (Dai et al., 2012; Gou et al., 2024); (2) stress responses: activation of the lignin biosynthesis pathway (Xu et al., 2020) and remodeling of the dynamic balance between hormone signaling and defense-related gene expression (Uji et al., 2024), which can enhance host resistance to biotic and abiotic stresses. Therefore, systematic analyses of functional networks and the regulation of MYB transcription factors may provide a theoretical basis and molecular targets for the genetic improvement of rice.

## 3 MYB transcription factors regulate rice growth and development

**Figure 2:**
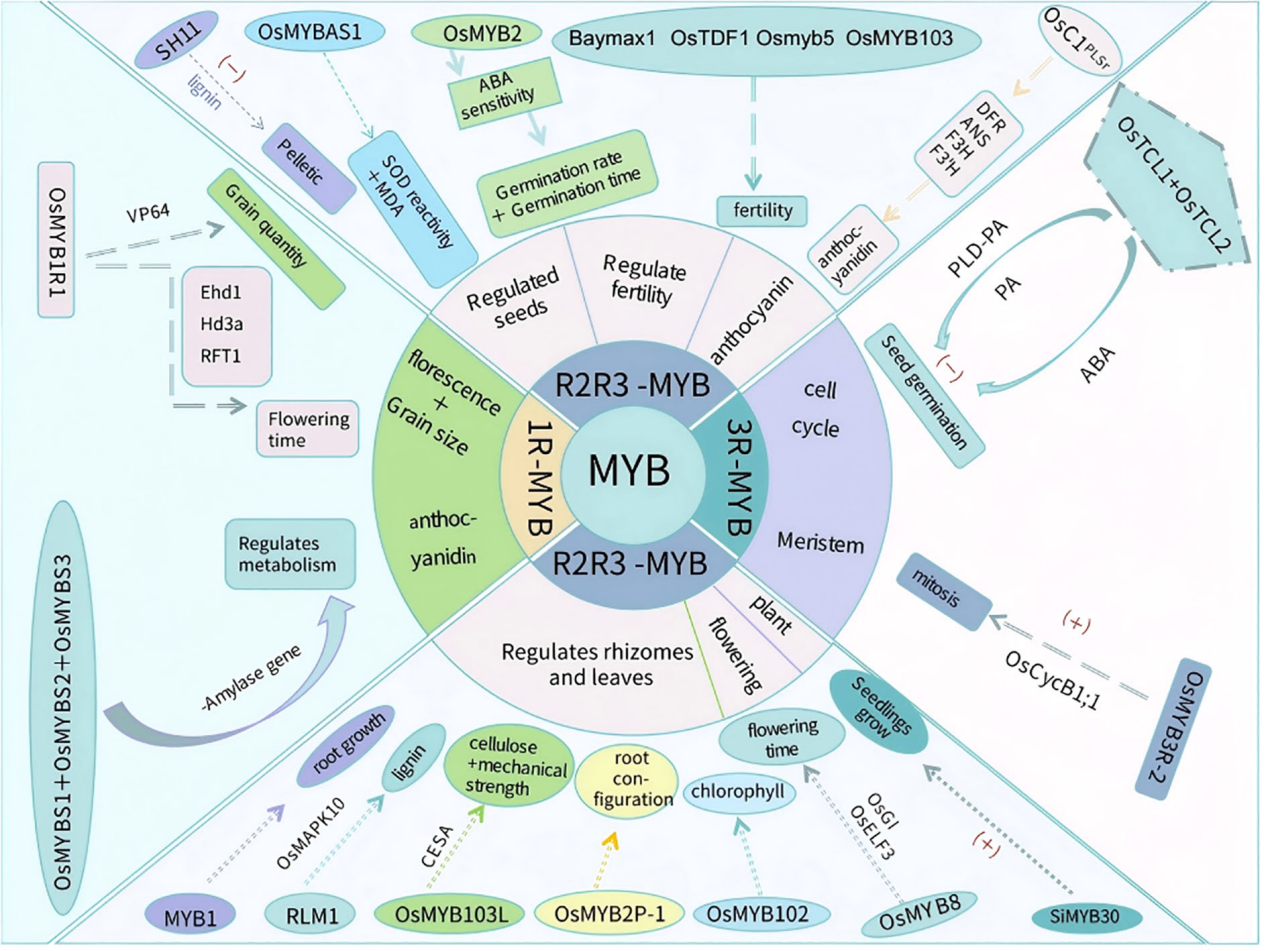
MYB transcription factors regulate rice growth and development. MYB transcription factors involved in regulating rice growth and development discussed in this paper are presented. These transcription factors are classified into three categories: 1R-MYB, R2R3-MYB, and 3R-MYB. The 1R-MYB transcription factor OsMYB1R1 can delay the flowering time and increase seed yield; OsMYBS1, OsMYBS2, and OsMYBS3 can regulate metabolism. Among the R2R3-MYB transcription factors, SH11 and OsMYBAS1 have regulatory effects on seeds; OsMYB2 decreases the seed germination rate and prolongs the germination time; Baymax1, OsTDF1, Osmyb5, and OsMYB103 decrease fertility; OsC1^PLSr^ promotes anthocyanin accumulation; MYB1, RLM1, OsMYB102, OsMYB103L, and OsMYB2P-1 regulate root, stem, and leaf development; OsMYB8 regulates the flowering time; and SiMYB30 promotes rice seedling growth. The 3R-MYB transcription factors OsTCL1 and OsTCL2 inhibit seed germination, whereas OsMYB3R-2 promotes mitosis. “+” indicates promotion; “−” indicates inhibition.

Different classes of MYB transcription factors have diverse functions related to rice growth and development (**Figure 2**). For example, 1R-MYB transcription factors mainly influence metabolic regulation and yield formation; R2R3-MYB transcription factors affect seed germination, tissue development (e.g., root, stem, and leaf), and fertility; and 3R-MYB transcription factors mainly regulate the cell cycle and seed germination. Notably, 4R-MYB transcription factors are relatively rare in plants, with functions that will need to be further analyzed.

### 3.1 MYB transcription factors regulate rice seed germination and grain drop

The rice life cycle from seed germination to grain drop is relatively long, with some MYB transcription factors (mainly R2R3-MYBs) playing important regulatory roles during this process (**Table 1**). 1R-MYB transcription factors regulate sugar metabolism, R2R3-MYB transcription factors control the antioxidant system and lignin synthesis pathway, and 3R-MYB transcription factors regulate the PLD/PA signaling pathway.

**Table 1:** Regulation of rice growth and development by different MYB transcription factors.

Types of MYB	Gene name	MSU_Locus	Function	References
1R-MYB	OsMYB1-R1	LOC_Os04g49450	Regulate grain yield	(Duan et al., 2014)
	OsMYBS 1	−	Involved in the sugar regulation of α-amylase	(Lu et al., 2002)
	OsMYBS 2	−	Involved in the sugar regulation of α-amylase	(Lu et al., 2002)
	OsMYBS 3	−	Involved in the sugar regulation of α-amylase	(Lu et al., 2002)
R2R3-MYB	MYB1	LOC_Os05g35500	Regulates root growth	(Gu et al., 2017)
	OsMYBAS1	LOC_Os11g47460	Promote deep seed sowing, germination and seedling formation	(Wang et al., 2022)
	OsMYB2P-1	LOC_Os05g04820	Regulates root configuration	(Dai et al., 2012)
	OsTDF1	LOC_Os03g18480	Regulates male fertility	(Cai et al., 2015)
	Baymax1	−	Regulates male fertility	(Xiang et al., 2021)
	OsC1	LOC_Os06g10350	Regulates anthocyanin synthesis	(Upadhyaya et al., 2021)
	OsC1^PLSr^	LOC_Os05g48010	Involved in the synthesis of anthocyanins	(Zou et al., 2023)
	SH11	−	Promotes seed fall	(Ning et al., 2023)
	RLM1	LOC_Os05g46610	Regulates the expression of key genes for lignin synthesis	(Chen et al., 2022a)
R2R3-MYB	MOF1	−	Affects rice spikelet development	(Ren et al., 2020)
	MFS2	−	Affects rice spikelet development	(Li et al., 2020)
	OsMYB2	LOC_Os03g20090	Change the sensitivity of seed germination	(Yang et al., 2012)
	Osmyb5	LOC_Os05g41166	Regulate rice fertility	(Wang et al., 2021)
	OsMYB8	LOC_Os01g45090	Regulate the flowering time	(Gou et al., 2024)
	SiMYB 30	−	Promote seedling growth	(Zhang et al., 2023)
	MYB61	LOC_Os01g18240	Regulates cellulose biosynthesis	(Gao et al., 2020)
	OsMYB102	LOC_Os06g43090	Delays leaf senescence	(Piao et al., 2019)
	OsMYB103L	LOC_Os08g05520	Reduces cellulose synthesis and mechanical strength of leaves	(Yang et al., 2014a)
	OsMYB103	LOC_Os04g39470	Regulate rice fertility	(Cai et al., 2015)
3R-MYB	OsTCL1	LOC_Os01g43180	Inhibits seed germination	(Yi et al., 2023)
	OsTCL2	LOC_Os01g43230	Inhibits seed germination	(Yi et al., 2023)
	OsMYB3R-2	LOC_Os01g62410	Regulates the cell cycle	(Ma et al., 2009)

“−” indicates none.

In rice, three 1R-MYBs (*OsMYBS1*, *OsMYBS2*, and *OsMYBS3*) are important for the regulation of sugar metabolism by α-amylase, which is produced in large quantities during seed germination to convert starch stored in the endosperm to energy required for seedling growth. According to earlier research, OsMYBS1 is a strong transcriptional activator that significantly suppresses the inhibitory effect of sugar on α-amylase gene expression, thereby decreasing starch decomposition and maintaining glucose metabolism homeostasis (Lu et al., 2002). By contrast, OsMYBS2 is a weak transcriptional activator that slightly enhances *α-amylase* gene expression, mainly in the presence of glucose, while OsMYBS3 may function as a transcriptional repressor that inhibits *α-amylase* gene expression in the absence of glucose.

OsMYBAS1 is an R2R3-MYB transcription factor that functions cooperatively with multiple molecular mechanisms to increase the rice seed germination rate and vigor under normal and stress conditions. A previous study isolated *OSMYBAS1* and determined that overexpressing this gene in transgenic rice plants can alleviate oxidative stress caused by deep sowing by enhancing the antioxidant system (Wang et al., 2022). Overexpressing another R2R3-MYB transcription factor OsMYB2 can significantly alter the sensitivity of seeds to ABA (Yang et al., 2012). Specifically, seed sensitivity to ABA is reportedly significantly higher for *OsMYB2*-overexpressing lines than for wild-type and RNAi lines; this increased sensitivity decreases the seed germination rate, prolongs the germination time, decreases seed vigor, and inhibits seed germination under stress conditions. Subsequent research demonstrated that the R2R3-MYB transcription factor SH11 adversely affects the expression of lignin synthesis-related genes, restricts lignin accumulation at the glume shell and cob junction, and decreases the mechanical strength of delaminated cells, thereby promoting seed shedding and promoting grain drop (Ning et al., 2023).

OsTCL1 and OsTCL2 are 3R-MYB transcription factors that inhibit seed germination under abiotic stress conditions through PLD/PA signaling and ABA-mediated pathways, thereby increasing seed survival during exposure to stress (Yi et al., 2023). Moreover, mutations to OsTCL1 and OsTCL2 lead to delayed seed germination and the down-regulated transcription of several PLD-encoding genes, resulting in decreased PA production. This suggests that OsTCL1 and OsTCL2 regulate seed germination partly through the PLD/PA signaling pathway. In addition, the expression of genes related to ABA biosynthesis, metabolism, and signaling is altered by mutations to both OsTCL1 and OsTCL2. The associated effects on endogenous ABA levels and the ABA response help regulate seed germination. This finding reveals the dual regulatory mechanism of OsTCL1 and OsTCL2 in seed germination, while also providing an important theoretical basis for rice breeding. Thus, 1R-MYB, R2R3-MYB, and 3R-MYB transcription factors play important roles in rice germination and grain drop.

### 3.2 MYB transcription factors regulate rice root, stem, and leaf development

R2R3-MYB and 3R-MYB transcription factors are important contributors to rice root, stem, and leaf development. During rice root development, R2R3-MYB transcription factors help regulate rice root configuration, whereas 3R-MYB transcription factors regulate the cell cycle. In terms of rice stem development, R2R3-MYB transcription factors control the lignin synthesis pathway. By contrast, during rice leaf development, R2R3-MYB transcription factors regulate several pathways, including those associated with cellulose synthesis, anthocyanin biosynthesis, and ABA signaling.

The root system is an important plant organ with multiple functions, including nitrogen fixation, water and nutrient absorption, organic matter synthesis, and metabolite secretion (Chen et al., 2022b; Dai et al., 2012). Further research revealed that MYB1 regulates root growth by affecting primary root elongation in a Pi-dependent manner, while also regulating lateral root development in a Pi-independent manner (Gu et al., 2017). Another study showed that the 3R-MYB transcription factor OsMYB3R-2 is important for the G2/M phase transition of the cell cycle (Ma et al., 2009). Specifically, it induces cells to enter the G2/M phase by regulating the expression of the gene encoding the cell cycle protein OsCycB1;1, which enhances mitotic activity. Cells in root apical and stem apical meristematic tissues are actively dividing, with OsMYB3R-2 regulating the cyclic progression of meristematic tissue cells.

The rice stem is a multi-functional organ that integrates support, transport, reproduction, and storage. Its structural and physiological properties directly affect yield, stress resistance, and how efficiently resources are used. Secondary cell wall development is closely related to lignin deposition, which is critical for enhancing cell wall mechanical strength and stress resistance.

In rice, the R2R3-MYB transcription factor RLM1 regulates the expression of a key lignin synthesis-related gene (*OsCAD2*) and affects secondary cell wall development. In leaves, ectopic *RLM1* expression results in a curled leaf phenotype, with the encoded protein regulating lignin synthesis by binding to the *OsCAD2* promoter, thereby modulating secondary cell wall formation. In stems, RLM1 interacts with OsMAPK10 and positively regulates the lignin content (Chen et al., 2022a). Accordingly, RLM1 is a critical regulator of secondary cell wall development and lignin deposition in rice because of its effects on *OsCAD2* expression and its interaction with OsMAPK10. In terms of molecular regulatory mechanisms, MAPK phosphatase 1 (MKP1) controls vascular tissue lignification by modulating the phosphorylation status of the MAPK-mediated transcription factor MYB4. MYB4 negatively regulates vascular tissue lignification by inhibiting the lignin biosynthesis pathway (Lin et al., 2022).

The leaf is the main organ for photosynthesis in rice. Its morphological characteristics and physiological status are crucial determinants of plant growth and development. Delaying leaf senescence helps to maintain high chlorophyll contents and enzyme activities, which ensures strong photosynthetic activities and promotes the accumulation of photosynthetic products at the late growth stage. *OsMYB* genes are highly expressed in rice leaves (Katiyar et al., 2012), implying that they may encode important regulators of leaf development and functions.

OsMYB103L is a transcription factor that affects cellulose synthesis in rice by controlling the expression of cellulose synthase (*CESA*)-encoding genes, which in turn influences leaf mechanical strength, with important regulatory effects on leaf development and mechanical strength (Yang et al., 2014a). Another R2R3-MYB transcription factor, OsMYB102, is a key regulator of leaf senescence that inhibits ABA accumulation and signaling; *OsMYB102* expression decreases leaf chlorophyll contents through the associated modulation of chlorophyll degradation-related gene expression (Piao et al., 2019).

Anthocyanins, an important class of plant pigments, are responsible for the vivid red and blue coloration of fruits and flowers. Their synthesis is precisely regulated by a variety of signaling pathways (Mol et al., 1996). In rice, the R2R3-MYB transcription factor OsC1 regulates anthocyanin biosynthesis to alleviate oxidative stress (Upadhyaya et al., 2021). The transcription factor OsC1^PLSr^ promotes anthocyanin biosynthesis by regulating the expression of key anthocyanin synthesis pathway genes encoding anthocyanin synthase *(ANS)*, dihydroflavonol reductase *(DFR)*, flavanone 3-hydroxylase *(F3H)*, and flavonoid 3′-hydroxylase *(F3′H)*, thereby enabling the accumulation of anthocyanins in leaf sheaths (Zou et al., 2023). Activated OsC1^PLSr^ up-regulates the expression of genes encoding *DFR, ANS, F3H, and F3′H* and mediates anthocyanin accumulation in H93S. In summary, different types of MYB transcription factors play diverse roles in the development of rice roots, stems, and leaves.

### 3.3 MYB transcription factors regulate rice flower spike development and yield

Research on the mechanisms regulating flower and panicle development is important for improving the yield and quality of cereal crops. Rice spikelets have a unique inflorescence structure, but the regulatory network underlying their development has not been thoroughly investigated. In-depth analyses of the molecular mechanisms controlling spikelet development may lead to new strategies for improving grain yield. 1R-MYB transcription factors regulate the nutritive growth period to improve grain yields, while R2R3-MYB transcription factors regulate the diurnal flowering time as well as spikelet and floral organ development.

The regulatory effects of an R2R3-MYB transcription factor (OsMYB8) and a jasmonate synthase (*OsJAR1*) on the rice diurnal floret opening time (DFOT) have been examined, which revealed that variations in *OsMYB8* between *indica* and *japonica* rice are significantly correlated with the observed diversity in flowering times (Gou et al., 2024). *OsMYB8* accelerates the flowering time by directly up-regulating the expression of downstream genes (e.g., *OsGI* and *OsELF3*), which affects the biological clock and photoperiodic response of rice. In addition, *OsMYB8* affects jasmonic acid–isoleucine biosynthesis and controls the diurnal floret opening time by directly or indirectly regulating *OsJAR1* expression.

More Floret1 (MOF1) encodes a MYB structural domain-containing protein with an amphiphilic repressor motif associated with a typical ethylene response factor; it is expressed in all rice organs and tissues. *MOF1* overexpression leads to an increase in the number of spikelets, whereas the RNAi-based knockdown of this gene has the opposite effect, indicating that MOF1 is a regulator of spikelet development. *MOF1* has genetic interactions with other spikelet development-related genes (e.g., *OsMADS1* and *OsMADS6*), which co-regulate spikelet formation (Ren et al., 2020). By contrast, Multi-Floret Spikelet 2 (MFS2) encodes a MYB transcription factor that interacts with OsTPR proteins through its C-terminal *EAR* motif to form a repressor complex, which regulates the expression of downstream genes and affects spikelet development (Li et al., 2020). MFS2 forms a complex with rice TPL/TPR proteins to repress the regulation of floral organ identity and spikelet meristem organization, with crucial effects on rice inflorescence development. *MFS2* overexpression alters the fate of the spikelet meristem and the production of multi-flowered spikelets, whereas the RNAi-based knockdown of this gene leads to a decrease in the number of floral organs. *MFS2* regulates the expression of downstream genes by directly binding to their promoter regions. Moreover, *MFS2* along with other spikelet and floral organ development-related genes (e.g., *OsMADS1* and *OsMADS6*) coordinately regulate spikelet and floral organ development.

The effects of *OsMYB1R1-VP64* on rice growth and development and yield have been clarified on the basis of transgenic rice plants overexpressing *OsMYB1R1-VP64* and several studies involving analyses of phenotypes, gene expression, and molecular mechanisms. The generated data indicate that the 1R-MYB transcription factor OsMYB1R1 regulates the seed yield mainly by prolonging the nutritive growth period (Wang et al., 2016). *OsMYB1R1* delays the flowering time of transgenic plants by down-regulating the expression of flowering-related genes, including *Ehd1*, *Hd3a*, and *RFT1*. When *OsMYB1R1* is overexpressed to produce a VP64-tagged fusion protein, it can significantly increase the seed yield, reflecting the important regulatory effects of OsMYB1R1 on rice growth and development and yield formation. In addition, specific alleles of *MYB61* promote biomass accumulation and increase the grain yield (Gao et al., 2020). The overexpression of the R2R3-MYB transcription factor gene *SiMYB30* can significantly increase above-ground fresh and dry weights, plant height, and root surface area at the seedling stage (Zhang et al., 2023).

These findings indicate that MYB transcription factors regulate rice growth, development, and stress responses at multiple molecular levels, while also providing an important theoretical basis and genetic resources for rice breeding.

### 3.4 MYB transcription factors regulate rice fertility

The mining and functional analysis of male fertility-related genes provide important insights and genetic resources relevant to improving rice production. In terms of their regulatory effects on rice fertility, R2R3-MYB transcription factors mainly modulate tapetum development. Earlier research showed that knocking down or down-regulating the expression of *OsTDF1* and *OsMYB103*, which encode R2R3-MYB transcription factors in rice, results in male sterility. In rice, OsMYB103 functions in the same genetic pathway as *OsTDF1*; the knockdown or down-regulation of *OsMYB103* expression leads to male sterility (Cai et al., 2015). *OsTDF1* regulates expression by binding directly to the promoter region of downstream genes, thereby affecting the development of the tapetum. *OsMYB103* overexpression leads to the abnormal development of the tapetum., whereas the RNAi-based knockdown of this gene results in the incomplete degradation of the tapetum. and decreased pollen fertility.

The R2R3-MYB transcription factor Baymax1 (BM1) plays a key role in the regulation of male fertility in rice. Notably, bm1 mutants have an abnormal anther chorionic layer, degraded mid-layer, and defective microspores; the resulting defective synthesis of the Ubisch body, abnormal formation of the outer wall of pollen, and microspore abortion lead to complete male sterility. BM1 may function as a homodimer that interacts with *EAT1*, *TIP2*, and *TIP3* to jointly regulate the development of the rice downy layer and microspores (Xiang et al., 2021).

Another important MYB transcription factor, CSA2 (Osmyb5), along with CSA regulates rice male fertility under both short- and long-day conditions (Wang et al., 2021). A loss-of-function mutation to CSA2 or the aberrant expression of this gene leads to the formation of a pollen outer wall with structural defects that affect pollen viability and fertility (e.g., decreased pollen germination rate), eventually leading to male sterility. CSA2 is especially important for pollen fertility under long-day conditions, serving as a key regulator of the transport and deposition of assimilates during the late pollen development stage. CSA and CSA2 control anther development in rice by regulating the same downstream genes. These findings provide an important theoretical basis and genetic resources for the functional analysis of male fertility-related genes in rice.

In summary, different classes of MYB transcription factors, but especially R2R3-MYBs, have important functions related to rice growth and development as well as metabolic regulation involving a complex molecular network. In-depth analyses of MYB transcription factor functions and their regulatory mechanisms may help to optimize rice growth and development as well as yield (**Table 1**).

## 4 Role of MYB transcription factors in abiotic stress responses

**Figure 3:**
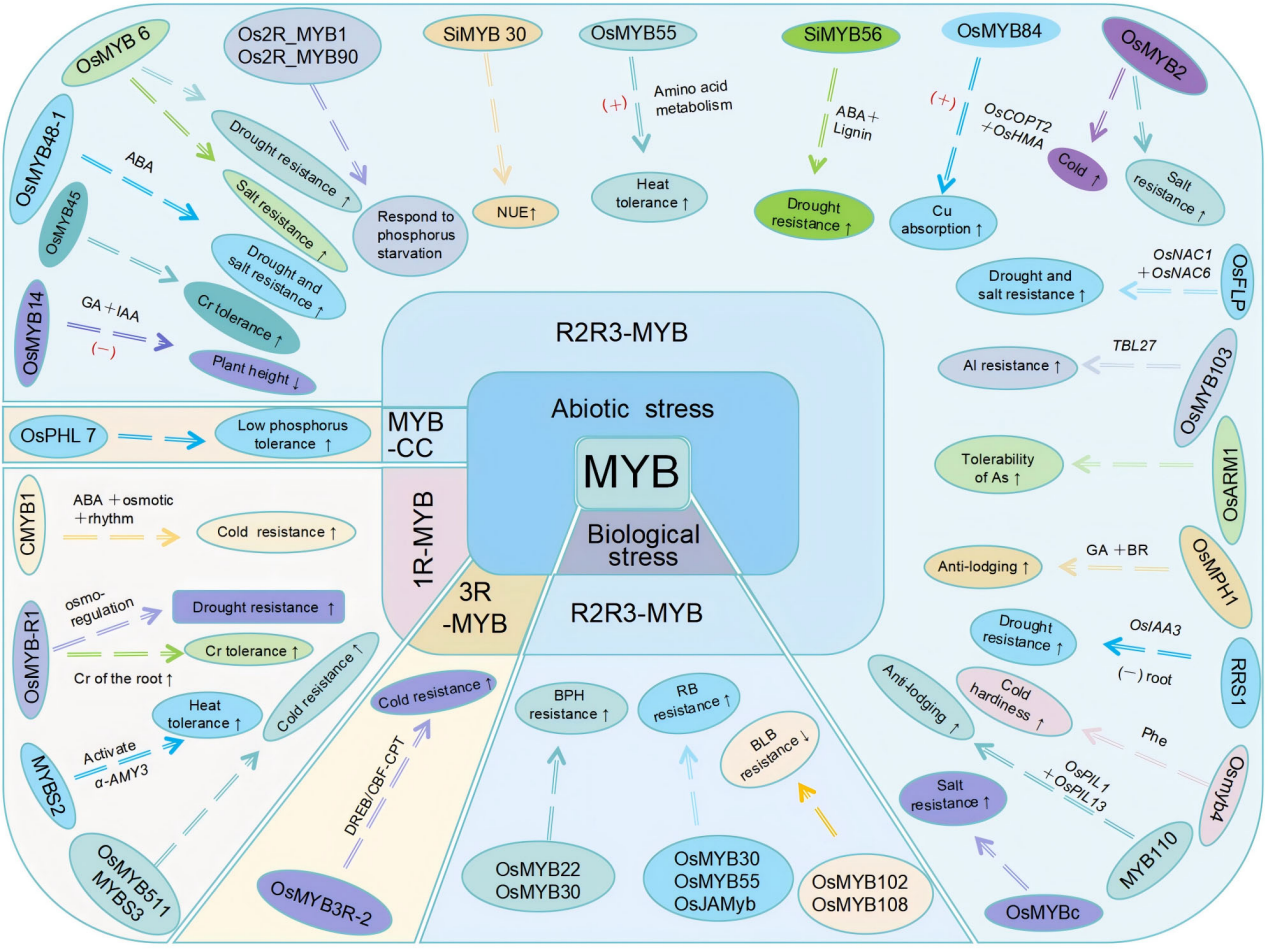
Responses of rice MYB transcription factors to biotic and abiotic stresses. Responses of MYB transcription factors to biotic and abiotic stresses discussed in this paper are presented. Among the transcription factors involved in abiotic stress responses, CMYB1 (1R-MYB transcription factor) enhances drought resistance; OsMYB-R1 enhances drought resistance and chromium stress tolerance; MYBS2 enhances heat resistance; and MYBS3 and OsMYB511 enhance cold resistance. OsPHL7 (MYB-CC family member) enhances low-phosphorus stress tolerance. Among the R2R3-MYB transcription factors, OsMYB14 negatively regulates plant height; OsMYB45 enhances cadmium stress tolerance; OsMYB48–1 enhances drought and salt stress tolerance; OsMYB6 enhances drought and salt stress tolerance; Os2R_MYB1 and Os2R_MYB90 are involved in phosphorus starvation signal transduction; SiMYB30 improves nitrogen use efficiency (NUE); OsMYB55 enhances high-temperature tolerance; SiMYB56 enhances drought resistance; OsMYB84 positively regulates copper absorption and transport; OsMYB2 enhances salt and cold stress tolerance. OsFLP enhances drought and salt stress tolerance; OsMYB103 enhances aluminum resistance; OsARM1 enhances arsenic tolerance; OsMPH1 improves lodging resistance; RRS1 enhances drought tolerance; Osmyb4 enhances low-temperature stress tolerance; MYB110 enhances lodging resistance; OsMYBc improves salt tolerance. The 3R-MYB transcription factor OsMYB3R-2 enhances cold tolerance. Among the transcription factors involved in biotic stress responses, the R2R3-MYB transcription factors OsMYB22 and OsMYB30 positively regulate BPH resistance; OsMYB30, OsMYB55, and OsJAMyb enhance rice blast disease resistance; and OsMYB102 and OsMYB108 decrease bacterial leaf blight resistance. “+” indicates activation/promotion; “−” indicates inhibition.

Abiotic stress significantly affects plant growth and crop productivity, with temperature, water, soil chemical, and mechanical stressors being the most common abiotic factors influencing agricultural production (Xiong et al., 2002). MYB transcription factors play key roles in stress responses. For example, 1R-MYB and R3-MYB types enhance rice cold tolerance, while R2R3-MYB types contribute to various abiotic stress responses (**Figure 3**).

### 4.1 Role of MYB transcription factors in response to temperature stress

Under high-temperature stress conditions, 1R-MYB and R2R3-MYB transcription factors have diverse functions (**Table 2**). Although both transcription factor types positively affect heat tolerance, 1R-MYB transcription factors activate stress-responsive genes, while R2R3-MYB transcription factors modulate the expression of genes associated with amino acid metabolism.

**Table 2:** Abiotic stress responses involving different MYB transcription factor.

Types of MYB	Gene name	MSU_Locus	Function	Downstream target genes	Governance mode	References
1R-MYB	CMYB1	LOC_Os02g46030	Resistant to low temperatures	−	+	(Duan et al., 2014)
	OsMYB-R1	LOC_Os04g49450	Drought resistance	OsP5CS1, OsProt, OsLEA3, OsRab16	+	(Yin et al., 2017)
			Resistant to heavy metals	−	+	(Tiwari et al., 2020)
	OsMYBS2	LOC_Os10g41260	Resistant to high temperatures	αAmy3, aAmy7	−	(Chen et al., 2019)
			Resistant to waterlogging	αAmy3	−	(Chen et al., 2019)
	OsMYBS3	LOC_Os10g41200	Resistant to low temperatures	DREB1	+	(Su et al., 2010)
	OsMYB511	−	Resistant to low temperatures	−	+	(Huang et al., 2015)
R2R3-MYB	Os2R_MYB1	−	Resistant to nutrient deficiencies	−	+	(Gu et al., 2017)
	OsARM1	LOC_Os05g37060	Resistant to heavy metals	OsLsi1, OsLsi2, OsLsi6	+	(Wang et al., 2017)
	OsMPH1	LOC_Os06g45890	Lodging resistance	−	+	(Zhang et al., 2017)
	OsRRS1	LOC_Os04g50770	Drought resistance	OsIAA3	+	(Gao et al., 2023a)
	OsMYB2	LOC_Os03g20090	Resistant to low temperatures	OsLEA3, OsRab16A, OsDREB2A	+	(Yang et al., 2012)
R2R3-MYB		LOC_Os12g07640	Salt resistant	OsLEA3, OsRab16A, OsDREB2A	+	(Yang et al., 2012)
	Osmyb4	LOC_Os01g50110	Resistant to low temperatures	−	+	(Vannini et al., 2004)
	OsMYB 6	LOC_Os04g58020	Drought resistance	−	+	(Tang et al., 2019)
			Salt resistant	−	+	(Tang et al., 2019)
	OsMYB14	−	Lodging resistance	OsGA20ox1, OsGA20ox2	−	(Kim et al., 2024)
	SiMYB16	−	Salt resistant	−	+	(Yu et al., 2023)
	SiMYB 30	−	Resistant to nutrient deficiencies	OsGOGAT2, OsGOGAT1, OsNIA2, OsNRT1, OsNRT1.1B	+	(Zhang et al., 2023)
	OsMYB30	LOC_Os02g41510	Resistant to low temperatures	−	−	(Lv et al., 2017)
		−	Resistant to heavy metals	Os4CL5	+	(Gao et al., 2023b)
	OsMYB45	LOC_Os06g45890	Resistant to heavy metals	−	+	(Hu et al., 2017)
	OsMYB48-1	−	Drought resistance	OsNCED4, OsNCED5	−	(Xiong et al., 2014)
	OsMYB55	LOC_Os05g48010	Resistant to high temperatures	OsGS1, GAT1, GAD3	+	(El-Kereamy et al., 2012)
	SiMYB56	−	Drought resistance	−	+	(Xu et al., 2020)
	OsMYBR57	LOC_Os06g08290	Drought resistance	OsLEA3, Rab21	+	(Yang et al., 2022)
R2R3-MYB	OsMYB60	LOC_Os12g03150	Drought resistance	OsCER1	+	(Jian et al., 2022)
	OsMYB84	LOC_Os03g56090	Resistant to nutrient deficiencies	OsCOPT2, OsHMA	+	(Ding et al., 2024)
	Os2R_MYB90	−	Resistant to nutrient deficiencies	−	−	(Yang et al., 2014b)
	OsMYB91	LOC_Os12g38400	Drought resistance	−	−	(Zhu et al., 2015)
			Salt resistant	SLR1	+	(Zhu et al., 2015)
			Lodging resistance	−	−	(Zhu et al., 2015)
	OsMYB103	LOC_Os04g39470	Resistant to heavy metals	−	+	(Wu et al., 2022)
	MYB110	−	Lodging resistance	OsPIL1, OsPIL13	−	(Wang et al., 2024)
	OsFLP	LOC_Os07g43420	Drought resistance	OsNAC1, OsNAC6	+	(Qu et al., 2022)
	OsMYBc	LOC_Os09g12770	Salt resistant	OsHKT1; 1	+	(Xiao et al., 2022)
3R-MYB	OsMYB3R-2	LOC_Os01g62410	Resistant to low temperatures	OsCycB1;1, OsCycB2;1,, OsCycB2;2, OsCDC20.1	+	(Ma et al., 2009)
MYB-CC	OsPHL 7	−	Resistant to nutrient deficiencies	−	−	(Yang et al., 2024)

“+” means positive regulation; “-” means negative regulation; “−” indicates none.

Global warming has resulted in decreased rice yields primarily because of the adverse effects of excessive heat during the temperature-sensitive flowering stage (Jagadish et al., 2010). Higher nighttime temperatures also contribute to yield losses (Peng et al., 2004). An exposure to heat stress during the early grouting period in southern China reportedly severely affects yield and quality (Dou et al., 2024). In response to high-temperature stress, α-Amy3 production in rice is activated via the functional inhibition of the 1R-MYB transcription factor MYBS2, leading to enhanced heat tolerance (Chen et al., 2019). In rice, the R2R3-MYB transcription factor OsMYB55 is a key regulator of high-temperature tolerance. Specifically, *OsMYB55* expression is induced under high-temperature stress conditions. Its overexpression in plants exposed to excessive heat promotes growth and helps maintain grain yields. *OsMYB55* directly induces the expression of several genes, including those encoding glutamine synthetase (*OsGS1;2*), glutamine aminotransferase (*GAT1)*, and glutamic acid decarboxylase 3 (*GAD3*), by binding to their promoter regions. This leads to an increase in the total amino acid content, with notable increases in L-glutamic acid, γ-aminobutyric acid, and arginine levels at high temperatures (El-Kereamy et al., 2012).

Low-temperature stress limits rice growth and productivity. Different MYB types participate in cold response-related pathways. 1R-MYB transcription factors affect sugar metabolism and osmoregulation, R2R3-MYB transcription factors influence amino acid and phenylalanine metabolism, and 3R-MYB transcription factors modulate cell-cycle regulation. Some MYBs, including OsMYB55, OsMYB511, and OsMYB1, act synergistically via shared target genes, protein interactions, and signaling cascades, thereby enhancing plant defense responses to cold stress at multiple levels.

Previous research showed that the 1R-MYB transcription factors CMYB1, MYBS3, and OsMYB511 positively affect rice resistance to low temperatures. Several genes encoding 1R-MYB transcription factors also improve cold resistance. CMYB1 regulates the expression of downstream cold response-related genes via ABA signaling, osmoregulation, and circadian pathways (Duan et al., 2014). MYBS3 was identified as a key cold-responsive regulator that represses the production of *DREB1* and *α-Amy3* SRC (Starch Repression Complex) at 4°C (Su et al., 2010). *OsMYB511* overexpression increases cold tolerance, whereas the silencing of this gene has the opposite effect (Huang et al., 2015).

*OsMYB2* expression is up-regulated under cold, salt, and dehydration stress conditions (Yang et al., 2012). *Osmyb4* expression increases at low temperatures, enhancing cold tolerance through phenylalanine metabolism and osmolyte biosynthesis (Vannini et al., 2004). *OsMYB30* negatively regulates cold tolerance by interacting with *OsJAZ9* and inhibiting *MYB* expression (Lv et al., 2017).

The 3R-MYB transcription factor OsMYB3R-2 binds to mitotic activator elements in cyclin gene promoters and increases proline levels via the DREB/CBF-CPT pathway, leading to enhanced cold tolerance (Ma et al., 2009). These studies revealed a multi-layered regulatory network involving MYB transcription factors that protect rice plants from cold stress. Hence, different MYB transcription factors play crucial roles in rice adaptive responses to temperature stress.

### 4.2 Role of MYB transcription factors in response to water stress

Waterlogging stress significantly affects rice growth. While moderate waterlogging may be beneficial, excessive flooding causes root hypoxia, stunts growth, reduces yield, and promotes disease, thereby severely limiting rice production. In response to waterlogging, 1R-MYB transcription factors protect rice plants by regulating sugar metabolism, antioxidant enzyme activities, photosynthetic efficiency, and energy metabolism.

Published research showed that the 1R-MYB transcription factor MYBS2 plays a key role in plant responses to submergence. Decreased *MYBS2* expression or increased *α-Amy3* expression improves plant tolerance to osmotic and drought stresses. Moreover, although submergence tolerance is adversely affected by *MYBS2* overexpression, it is not enhanced by knocking down *MYBS2* expression (Chen et al., 2019). MYBS2 helps maintain growth under flooding conditions by regulating sugar metabolism and the energy balance. It serves as a sugar starvation-responsive transcription factor that alters the expression of sugar-related genes and helps plants adapt to low-sugar conditions. It also enhances flooding tolerance by modulating antioxidant activities, photosynthesis, and energy use. These observations reflect the complex roles of 1R-MYB transcription factors that contribute to waterlogging tolerance.

Drought is another major abiotic stress that impairs plant growth by limiting water uptake (Li et al., 2019). Rice is particularly sensitive to drought stress (Huang et al., 2014). 1R-MYB transcription factors influence osmoregulation under drought conditions, whereas R2R3-MYB types regulate stomatal opening, root development, wax synthesis, antioxidant defense, and ABA signaling. The 1R-MYB transcription factor OsMYBR1 increases drought tolerance and decreases ABA sensitivity by regulating osmoregulatory substances and the expression of stress-responsive genes (Yin et al., 2017).

OsMYB6 (R2R3-MYB) enhances drought tolerance through its effects on osmolytes and antioxidant systems. *OsMYB6* overexpression increases the proline content as well as CAT (Catalase) and SOD (Superoxide Dismutase) activities, while decreasing REL (Relative Electrolyte Leakage) and MDA (Malondialdehyde) levels under stress conditions (Tang et al., 2019). OsMYB48-1 (R2R3-MYB) improves drought and salt tolerance by regulating the expression of ABA biosynthesis-related genes (e.g., *OsNCED4* and *OsNCED5*), early signaling genes (e.g., *OsPP2C68* and *OSRK1*), and late-response genes (e.g., *RAB21* and *OsLEA3*) (Xiong et al., 2014). Similarly, SiMYB56 improves drought tolerance in transgenic rice plants by altering lignin biosynthesis, regulating the ABA pathway, and increasing ABA accumulation and the expression of stress-responsive genes (Xu et al., 2020). OsMYB60 promotes leaf wax biosynthesis by activating the expression of a key wax-related gene (*OsCER1*). Its loss reportedly leads to decreases in the wax content and drought tolerance (Jian et al., 2022). *OsMYB91* expression is induced under drought conditions, but the overexpression of this gene results in defective growth (Zhu et al., 2015), indicating that the encoded transcription factor has a complex role. By contrast, OsFLP improves drought and salt tolerance by activating the expression of *OsNAC1* and *OsNAC6*, which encode regulators of stomatal opening and the expression of stress-responsive genes (e.g., *DST*, *OsLEA3*, and *OsDREB2A*) (Qu et al., 2022).

Root system development is critical for water uptake. RRS1, which is an R2R3-MYB transcription factor, negatively regulates root growth. Silencing *RRS1* expression increases primary and lateral root lengths and densities, with positive effects on drought tolerance (Gao et al., 2023a). OsMYBR57 directly regulates the expression of several drought-related *OsbZIP* genes. Additionally, the OsFTIP6*–*OsHB22–OsMYBR57 module mediates drought response mechanisms. Specifically, *OsMYBR57* interacts with *OsHB22*, while *OsFTIP6* promotes the nuclear shuttling of *OsHB22*, resulting in the activation of drought-responsive genes (e.g., *OsLEA3* and *Rab21*) (Yang et al., 2022). These studies revealed diverse mechanisms involving MYB transcription factors that mediate rice plant responses to drought, providing important insights into rice drought resistance-related molecular networks.

### 4.3 Role of MYB transcription factors in response to soil chemical stress

Soil salinization severely limits agricultural productivity and threatens rice production, potentially leading to food insecurity and socioeconomic challenges (Ismail and Horie, 2017). Plants have evolved molecular, cellular, and physiological adaptive mechanisms to cope with high-salt conditions (Ma et al., 2022). As a salt-sensitive crop, rice is highly vulnerable to salinity stress, which can impair its growth and decrease yields. Therefore, cloning salt tolerance-related genes and breeding tolerant varieties are key strategies for improving rice production and sustainable development.

In rice, R2R3-MYB transcription factors regulate responses to salt stress through ubiquitination, lignin and suberin biosynthesis, and ABA signaling. Sodium–potassium homeostasis is critical in saline soils. The expression of *OsHKT1;1*, which encodes a high-affinity K^+^ transporter that regulates shoot Na^+^ exclusion, is controlled by the R2R3-MYB transcription factor OsMYBc. OsMSRFP ubiquitinates OsMYBc, which attenuates *OsHKT1;1* expression; its knockdown enhances salt tolerance. *OsMYBc*-overexpressing plants exhibit increased salt tolerance, revealing a ubiquitination-mediated regulatory mechanism (Xiao et al., 2022). *OsMYB2* and *OsMYB6* overexpression significantly improves salt tolerance (Yang et al., 2012; Tang et al., 2019). In *OsMYB2*-overexpressing plants, proline and soluble sugars accumulate under salt stress conditions and the expression levels of proline synthase and transporter genes are up-regulated. Similarly, the expression of stress-responsive genes (e.g., *OsLEA3*, *OsRab16A*, and *OsDREB2A*) is up-regulated. OsMYB6 enhances salt tolerance without affecting growth, but it increases proline, CAT, and SOD activities, while decreasing REL and MDA contents. Abiotic stress-responsive genes are highly expressed in transgenic plants. SiMYB16 enhances salt tolerance throughout the growth cycle by regulating lignin and suberin pathways (Yu et al., 2023). *OsMYB91* expression is induced by salt stress and ABA; its overexpression increases salt tolerance despite inhibiting growth (Zhu et al., 2015). The encoded transcription factor regulates *SLR1* expression in response to multiple stresses and hormones.

The main heavy metals in rice are chromium (Cr), aluminum (Al), and arsenic (As). MYB transcription factors (mainly 1R-MYBs and R2R3-MYBs) have important roles related to plant responses to heavy metal stress. 1R-MYB transcription factors regulate the accumulation and distribution of Cr in roots to protect rice from Cr stress. By contrast, R2R3-MYB transcription factors affect rice cell wall properties, regulate a signaling pathway upstream of *TBL27*, and bind to the promoter of As transporter-encoding genes (*OsLsi1*, *OsLsi2*, and *OsLsi6*). In terms of the effects of heavy metal stress, Cr is a toxic metallic element with carcinogenic and teratogenic properties.

Under heavy metal stress conditions, MYB transcription factors mitigate the toxic effects of Cr, Al, and As. The 1R-MYB transcription factor OsMYB-R1 improves Cr tolerance by increasing root Cr accumulation and GST activity (Tiwari et al., 2020). OsMYB45 enhances Cd tolerance via transcriptional regulation in roots (Hu et al., 2017). Notably, Al toxicity is a major cause of crop yield decreases and forest degradation in acidic soils (Kochian, 1995). Specifically, Al can inhibit crop root elongation, while also disrupting cell division and elongation zone development (Chauhan et al., 2021).

Previous research involving Arabidopsis showed that the R2R3-MYB transcription factor MYB103 regulates the O-acetylation of cell wall xyloglucans by controlling a signaling pathway upstream of *TBL27*, thereby increasing plant resistance to Al toxicity (Wu et al., 2022). In rice, ART1 confers resistance to Al toxicity by inhibiting the modification of cell wall properties mediated by the R2R3-MYB transcription factor OsMYB30 (Gao et al., 2023b). Earlier research demonstrated that putrescine inhibits *OsMYB30* expression by eliminating Al-induced H_2_O_2_ accumulation, whereas ART1 directly inhibits *OsMYB30* expression, which in turn decreases Os4CL5-dependent 4-coumaric acid accumulation and the Al-binding capacity of the cell wall, resulting in enhanced Al resistance.

As contamination poses a serious threat to rice production, particularly in heavily contaminated regions, and has become a major reason for dietary exposure to As in China (Li et al., 2011). The excessive accumulation of inorganic As in rice grains is a potential health risk for populations that consume rice as a staple food (Zhao et al., 2010). The R2R3-MYB transcription factor OsARM1 plays a key role in the rice response to As stress. Specifically, it regulates the expression of As transporter genes (*OsLsi1*, *OsLsi2*, and *OsLsi6*), thereby influencing As uptake and root-to-shoot translocation (Wang et al., 2017).

MYB transcription factors, including R2R3-MYB and MYB-CC types, are also involved in rice responses to nutrient deficiency. More specifically, they modulate phosphorus signaling, nitrogen metabolism, and root architecture. For instance, phosphorus deficiency inhibits growth-related processes such as leaf elongation (Kavanová et al., 2006). Given that phosphate resources are non-renewable and readily immobilized in soil, addressing phosphorus deficiency has become a major challenge in agricultural production. Research indicated that the PUE (P use Efficiency) in global crop production must be increased to 68–81% to meet the demands of sustainable food production (Zou et al., 2022). Therefore, enhancing phosphorus utilization is essential, underscoring the need to elucidate the molecular mechanisms underlying plant adaptations to low-phosphorus stress (Wasaki et al., 2003). Indeed, studies have demonstrated that plants have evolved a suite of physiological and molecular mechanisms to cope with low phosphorus availability (Wasaki et al., 2006; Li et al., 2010).

R2R3-MYB transcription factors contribute to nutrient stress responses by modifying the root system architecture, particularly under phosphorus starvation conditions (Chen et al., 2022b). For example, Os2R_MYB1 participates in phosphorus starvation-related signaling pathways (Gu et al., 2017), while Os2R_MYB90 regulates phosphorus accumulation and transporter gene expression (Yang et al., 2014b). Moreover, OsPHL7 (MYB-CC family member) enhances plant tolerance to low phosphorus levels. *OsPHL7*-overexpressing lines exhibit increased root biomass accumulation as well as elevated phosphorus contents in shoots and roots under normal and low-phosphorus conditions (Yang et al., 2024).

Nitrogen is the most important macronutrient element in plants because of its critical effects on growth and development (Lian et al., 2006). The R2R3-MYB transcription factor SiMYB30 increases nitrogen use efficiency by activating the expression of nitrogen metabolism-related genes (e.g., *OsGOGAT2*, *OsGOGAT1*, *OsNIA2*, *OsNRT1*, and *OsNRT1.1B*) to promote nitrogen uptake, transport, and assimilation (Zhang et al., 2023).

Deficiencies in trace elements, such as copper (Cu), can retard growth, reduce yield, and decrease grain quality. The R2R3-MYB transcription factor OsMYB84 positively regulates Cu uptake and transport by inducing the expression of Cu transporter genes (*OsCOPT2* and *OsHMA*) (Ding et al., 2024). *OsMYB84* overexpression improves Cu uptake, root-to-stem translocation, and grain Cu distribution, while also significantly increasing grain yields.

### 4.4 Role of MYB transcription factors in response to mechanical stress

Lodging stress is a form of mechanical stress. Rice plant height is a key agronomic trait that influences crop architecture, apical dominance, biomass, lodging resistance, crowding tolerance, and mechanized harvesting efficiency (Liu et al., 2018a). R2R3-MYB transcription factors regulate lodging resistance through hormone metabolic pathways, GA and brassinosteroid (BR) signaling, and cell wall biosynthesis, while also inhibiting cell expansion and modulating downstream gene expression. The R2R3-MYB transcription factor OsMYB14 affects plant height via hormone metabolism; *OsMYB14* overexpression results in dwarfism (Kim et al., 2024). OsMYB14 negatively regulates plant height through GA and auxin (IAA) pathways and may indirectly influence height via its effects on the expression of genes related to development and metabolism.

Under phosphorus starvation conditions, *MYB110*, which is a direct target of *OsPHR2*, negatively regulates plant height (Wang et al., 2024). It enhances lodging resistance by inhibiting cell expansion and regulating downstream targets, including *OsPIL1* and *OsPIL13*. Mutating or overexpressing *MYB110* can improve lodging resistance. Similarly, elevated *OsMYB91* expression levels can decrease cell elongation and plant height (Zhu et al., 2015). In addition, *OsMPH1* (Zhang et al., 2017) positively regulates plant height, lodging resistance, and yield by altering GA and BR signaling and cell wall synthesis. *OsMPH1* overexpression increases plant height and yield, but knocking down the expression of this gene has the opposite effects. OsMPH1 modulates the internode cell length, highlighting its effects on plant architecture. These studies demonstrate that R2R3-MYB transcription factors regulate plant height and lodging resistance through diverse mechanisms, providing a basis for the genetic improvement of rice plant types and stress resilience.

In summary, various MYB transcription factors play crucial roles in rice plant responses to abiotic stress involving complex regulatory networks, with R2R3-MYBs exhibiting the broadest functional diversity. Further research into the mechanisms underlying their functions will support the breeding of stress-resistant varieties and improve yield stability under adverse conditions.

## 5 Role of MYB transcription factors in response to biotic stress

Rice plants are susceptible to pest infestations and pathogen infections during growth and development, which severely restrict yield and quality formation. The MYB protein family, which is the largest family of transcription factors in plants, mainly consists of R2R3-MYB transcription factors that play a regulatory role in rice responses to biotic stresses. For example, these transcription factors contribute to rice defense responses to pathogens and pests by regulating the expression of downstream resistance-related genes, while also activating cinnamic acid/monosaccharide and lignin biosynthesis pathways and modulating hormone signaling pathways. More specifically, R2R3-MYB transcription factors recognize specific DNA sequences and activate or inhibit the expression of target genes, thereby regulating resistance-related physiological processes, including phytohormone signaling and the synthesis of secondary metabolites (**Table 3**).

**Table 3:** Biotic stress responses involving different MYB transcription factors.

Types of MYB	Gene name	MSU_Locus	Function	Downstream target genes	References
R2R3-MYB	OsMYB22	LOC_Os01g65370	Resistant to brown planthoppers	F3'H	(Sun et al., 2024)
	OsMYB30	LOC_Os09g26170	Resistant to brown planthoppers	OsPAL6, OsPAL8	(Sun et al., 2024)
	OsMYB30	LOC_Os02g41510	Resistant to rice blast	−	(Kishi-Kaboshi et al., 2018)
	OsMYB55	LOC_Os04g43680	Resistant to rice blast	Os4CL3, Os4CL5	(Kishi-Kaboshi et al., 2018)
	OsMYB102	LOC_Os06g43090	Resistant to white leaf blight	−	(Miyamoto et al., 2019; Lin et al., 2022)
	OsMYB108	LOC_Os09g36730	Resistant to white leaf blight	−	(Miyamoto et al., 2019; Lin et al., 2022)
	OsMYB110	LOC_Os10g33810	Resistant to rice blast	−	(Kishi-Kaboshi et al., 2018)
	OsJAMyb	LOC_Os11g45740	Resistant to rice blast	−	(Cao et al., 2015)
−	JMTF1	−	Resistant to white leaf blight	OsPrx26, OsTPS24	(Uji et al., 2024)

“−” indicates none.

### 5.1 Role of MYB transcription factors in rice blast disease resistance

Rice blast (also known as rice fever) caused by Magnaporthe oryzae is one of the most devastating diseases affecting global rice production (Li et al., 2016). The pathogen can infect rice throughout the reproductive period, causing severe damages to all plant parts and leading to widespread yield losses. R2R3-MYB transcription factors mediate pathogen defenses by directly regulating target gene expression, activating immunity-related morphological development, mediating plant hypersensitive reactions, and altering salicylic acid (SA) or jasmonic acid (JA) signaling.

The R2R3-MYB transcription factor OsJAMyb also enhances rice blast resistance (Cao et al., 2015). Moreover, a natural allele of a *C2H2*-type transcription factor gene encodes a protein that inhibits H_2_O_2_ degradation by regulating *bsr-d1* expression, thereby enhancing disease resistance (Li et al., 2016, 2017). This finding provides important clues regarding the molecular basis of rice blast resistance. In addition, the R2R3-MYB transcription factors OsMYB30, OsMYB55, and OsMYB110 positively regulate disease resistance. Furthermore, *MYB30*, *MYB55*, and *MYB110* activate the cinnamic acid/monosaccharide pathway, leading to the synthesis of lignin precursors and hydroxycinnamic acids, which are involved in the perception of microbe-associated molecular patterns. MYB transcription factors induce the accumulation of ferulic acid (a hydroxycinnamic acid) *in vivo* and enhance immune responses to protect rice from *M. oryzae* and the pathogen responsible for bacterial leaf blight (Kishi-Kaboshi et al., 2018).

### 5.2 Role of MYB transcription factors in rice resistance to bacterial leaf blight

Bacterial leaf blight caused by *Xanthomonas oryzae* pv. *oryzae* (Xoo) is the most devastating bacterial disease of rice worldwide. R2R3-MYB transcription factors contribute to rice disease resistance by regulating hormone signaling pathways and lignin biosynthesis. Two MYB4 homologs (*OsMYB102* and *OsMYB108*) in the rice genome encode negative regulators of lignin synthesis and leaf blight resistance. Specifically, they negatively regulate rice resistance to leaf blight through the MKP1–MPK3/6–MYB signaling cascade pathway (Miyamoto et al., 2019; Lin et al., 2022).

JMTF1 is a JA-responsive transcription factor that positively regulates rice resistance to Xoo by coordinating JA and growth hormone signaling pathways (Uji et al., 2024). Lines overexpressing *JMTF1* have a JA-hypersensitive phenotype and exhibit significantly enhanced disease resistance. The underlying mechanism involves the up-regulated expression of the peroxidase gene *OsPrx26* and the monoterpene synthase gene *OsTPS24*. Transgenic rice plants overexpressing *OsPrx26* exhibit enhanced resistance to Xoo, with down-regulated expression of auxin-responsive genes and up-regulated expression of the auxin signaling repressor gene *OsIAA13*. Gain-of-function mutations to *OsIAA13* positively affect Xoo resistance. JMTF1 selectively binds to the *OsPrx26* promoter region *in vivo*.

### 5.3 Role of MYB transcription factors in rice resistance to brown planthopper infestations

Pests are important biotic stressors affecting rice growth and development. The brown planthopper (BPH), a stinging insect endemic to rice, has been a major pest affecting rice cultivation in recent years. MicroRNAs are key regulators of plant–environment interactions. MYB transcription factors mediate rice resistance to BPH through multiple molecular mechanisms. Additionally, the OsmiR319–OsPCF5 regulatory module plays an important role in rice resistance to BPH; *OsPCF5* is targeted by *OsmiR319* as well as by various R2R3-MYB transcription factors, including OsMYB22, OsMYB30, and OsMYB30C (Sun et al., 2024). In terms of defense mechanisms, the R2R3-MYB transcription factor OsMYB30 increases the phenylalanine ammonia-lyase (PAL) content and promotes lignin and SA accumulation by activating *OsPAL6* and *OsPAL8* transcription to protect rice plants from BPH infestations. Furthermore, *OsMYB30* directly up-regulates *OsPAL6* and *OsPAL8* expression levels (He et al., 2020).

### 5.4 Role of MYB transcription factors in rice resistance to stripe leaf blight

JA signaling during rice plant defense responses to stripe leaf blight enhances RNA silencing and disease resistance (Yang et al., 2020). Mechanistically, the JA-responsive transcription factor JAMYB binds directly to the *AGO18* promoter to activate transcription. The perception of the rice stripe virus (RSV) capsid protein (CP) triggers JA accumulation and up-regulates *JAMYB* expression, thereby initiating the host defense network. This mechanism reveals the synergistic roles of the JA signaling pathway and RNA silencing in rice antiviral defense responses.

In summary, R2R3-MYB transcription factors participate in a complex immune network mediating rice responses to biotic stresses via multiple pathways (e.g., hormone crosstalk, metabolic reprogramming, defense gene activation, and feedback signaling). In-depth investigations of their functions and regulatory mechanisms may further elucidate the molecular basis of rice stress tolerance, while also providing breeders with key genetic targets for developing new rice varieties with broad-spectrum resistance to pests and pathogens. Future research should focus on upstream signal perception-related mechanisms, protein interactions, and spatiotemporal dynamic regulation at the single-cell level to further advance crop disease resistance breeding programs.

## 6 Summary and outlook

By systematically reviewing research on rice MYB transcription factors over the past decade, we summarized their multi-dimensional regulatory effects on plant morphogenesis and stress responses (Wang et al., 2016; Chen et al., 2022a; Yi et al., 2023). Multiple MYB transcription factors have synergistic functions (Cai et al., 2015). As members of a plant-specific transcription factor family, MYB proteins dynamically regulate upstream and downstream gene expression networks, significantly influencing rice growth, development, and stress tolerance. In rice, MYB transcription factors serve as molecular hubs that integrate internal developmental signals with external stimuli. They link growth and development with stress responses, thereby balancing normal growth and adaptations to various stresses through dynamic gene regulation. This allows rice plants to remain viable and reproduce under complex conditions. Thus, MYB transcription factor genes represent key targets for genetic improvement, offering great potential for breeding high-yielding and stress-tolerant rice varieties (Liu et al., 2025). Current research on MYB transcription factors is increasingly applying network-based approaches and single-cell analytical methods with crop-specific applications and diverse utility. Future studies should integrate multi-dimensional technologies to decipher molecular mechanisms and accelerate field applications.

However, several challenges remain. For example, functional redundancy and signal integration-related mechanisms are still poorly understood. Individual MYB transcription factors often contribute to multiple stress responses. Hence, their dynamic regulatory networks under combined stress conditions require further investigation. Research paradigms are shifting from single-gene studies to systematic analyses of protein interaction networks; from tissue-level expression to single-cell resolution; and from mechanisms in Arabidopsis models to crop-specific mechanisms using various tools (e.g., CRISPR-Cas9 and multi-omics technologies) (Jinek et al., 2012). Furthermore, non-R2R3-MYB subfamilies (e.g., 1R, R3, and 4R-MYB) remain understudied in terms of their functions related to stress resistance. There is a particular deficiency in our understanding of plant responses to heavy metal stress and the cross-regulation of pest and pathogen resistance. To address these issues, future research should focus on the following:

1. CRISPR-Cas9 gene editing, multi-omics analyses, and 3D genome technologies (e.g., ATAC-seq and Hi-C) (Lieberman-Aiden et al., 2009; Jinek et al., 2012) to decipher MYB regulatory mechanisms under heavy metal stress conditions;
2. Protein interactomics (e.g., TurboID proximity labeling) (Branon et al., 2018), chromatin conformation capture (ChIA-PET), and liquid–liquid phase separation investigations to elucidate MYB transcription factor functions in transcriptional condensate formation and long-range gene regulation;
3. Rice pan-genome and genome-wide association studies to identify beneficial alleles and structural variants, with precise gene editing (e.g., Prime Editing) (Anzalone et al., 2019) and RNA regulatory systems (e.g., Cas13d) used to design smart stress tolerance modules for developing novel germplasm with stress resistance combined with high yields.

Concurrently, efforts are needed to assess whether laboratory findings are applicable to field-grown plants. Multi-location, multi-generation, and multi-environment trials must be conducted to fully evaluate the agronomic traits, stability, and stress tolerance of gene-edited lines under field conditions. The benefits of technological applications must be assessed and innovative promotion models should be explored (e.g., public–private partnerships) to ensure that smallholder farmers with limited resources can benefit from yield increases due to the findings of genetic research.

In summary, MYB transcription factors are pivotal molecular hubs that connect rice growth and development with stress responses. By integrating internal developmental signals and environmental cues, they dynamically regulate gene expression networks, enabling rice plants to grow normally and reproduce, while responding flexibly to environmental challenges. As key targets for genetic improvement, MYB transcription factor genes are potentially useful for breeding high-yielding and stress-tolerant rice varieties. Current research is moving toward network-based, single-cell, crop-specific, and diversified approaches. Future work must integrate multi-dimensional technologies to comprehensively characterize molecular mechanisms and promote the translation of basic research findings into field applications, which may lead to breakthroughs in next-generation crop breeding.
